# Does hysteroscopic resection of uterine septum improve reproductive outcomes: a systematic review and meta-analysis

**DOI:** 10.1007/s00404-021-05975-2

**Published:** 2021-02-07

**Authors:** Monica Krishnan, Brenda F. Narice, Bolarinde Ola, Mostafa Metwally

**Affiliations:** The Assisted Conception Unit, Sheffield Teaching Hospitals and the University of Sheffield, Sheffield, S10 2SF UK

**Keywords:** Uterine septum, Septum resection, Reproductive outcomes, Hysteroscopy

## Abstract

**Purpose:**

Uterine septum in women with subfertility or previous poor reproductive outcomes presents a clinical dilemma. Hysteroscopic septum resection has been previously associated with adverse reproductive outcomes but the evidence remains inconclusive. We aimed to thoroughly and systematically appraise relevant evidence on the impact of hysteroscopically resecting the uterine septum on this cohort of women.

**Methods:**

AMED, BNI, CINAHL, EMBASE, EMCARE, Medline, PsychInfo, PubMed, Cochrane register of controlled trials, Cochrane database of systematic reviews and CINAHL were assessed to April 2020, with no language restriction. Only randomised control trials and comparative studies which evaluated outcomes in women with uterine septum and a history of subfertility and/or poor reproductive outcomes treated by hysteroscopic septum resection against control were included. The primary endpoint was live birth rate, whereas clinical pregnancy, miscarriage, preterm birth and malpresentation rates were secondary outcomes.

**Results:**

Seven studies involving 407 women with hysteroscopic septum resection and 252 with conservative management were included in the meta-analysis. Hysteroscopic septum resection was associated with a lower rate of miscarriage (OR 0.25, 95% CI 0.07–0.88) compared with untreated women. No significant effect was seen on live birth, clinical pregnancy rate or preterm delivery. However, there were fewer malpresentations during labour in the treated group (OR 0.22, 95% CI 0.06–0.73).

**Conclusion:**

Our review found no significant effect of hysteroscopic resection on live birth. However, given the limited evidence available, high-quality randomised controlled trials are recommended before any conclusive clinical guidance can be drawn.

**Supplementary Information:**

The online version contains supplementary material available at 10.1007/s00404-021-05975-2.

## Introduction

Septate uterus is the most common Müllerian anomaly in women with an estimated incidence of 0.2–2.3%, subject to the diagnostic methods and classification system [1, 2]. It can be categorised into partial (subseptate) or complete septate groups [3] and is accountable for poor reproductive outcomes and obstetric problems, such as pregnancy loss, preterm birth and foetal malpresentations [1, 4]. The most commonly seen reproductive complication is spontaneous miscarriage, affecting more than 60% of women with uterine septum [5–7]. The existence of a uterine septum can frequently lead to habitual abortion, although some patients with uterine septum are asymptomatic and are able to conceive and deliver without struggle. The mechanism by which uterine septum causes pregnancy loss is not fully understood. It has been proposed that abnormal implantation dynamics caused by poor blood supply to the septum leads to spontaneous miscarriages [8–10]. Hence, it can be hypothesised that basis of treatment should be restoration of normal uterine cavity.

It has been suggested that uterine septum is a potential cause of infertility [7]. Many studies have described women with septate uterus with otherwise unexplained primary infertility; however, the role of uterine septum in infertility and the indications for uterine septum resection remains controversial [9, 11–14]. Standard treatment modality for uterine septum is through a hysteroscopic approach [15]. Although it is a relatively expeditious, efficient and safe method, it can be associated with complications that may adversely affect reproductive performance such as perforation and postoperative synechia.

The efficacy of hysteroscopic septum resection to improve reproductive outcomes remains unclear as no prospective randomised trials comparing hysteroscopic septum resection to no intervention have been published so far. The limited evidence available on the impact of hysteroscopic septum resection arises from studies which compare reproductive outcomes on the same group of patients before and after surgery [4, 9, 16]. While this approach can provide useful information on reproductive performance in women with uterine septum, it lacks the robustness of trials with independent matched treatment and control groups to properly assess the efficacy of hysteroscopic septum resection.

An added challenge to safely guide hysteroscopic septum resection practice in the clinical setting is the lack of consensus amongst experts on the classification of uterine anomalies. In 2013, the European Society of Human Reproduction and Embryology (ESHRE)/ the European Society for Gynaecological Endoscopy (ESGE) put together a new classification of uterine anomalies based on their anatomy for better consensus and to aid management [17]. The uterine anomalies were classified from U0 to U6. In this review we only looked at uterine anomaly classification U2 which are septate uterus.

The National Institute for Health and Care Excellence (NICE) and American Society of Reproductive Medicine (ASRM) support the performance of hysteroscopic resection providing it adheres to clinical governance, whereas the European Society of Human Reproduction and Embryology (ESHRE) and the Royal College of Obstetricians and Gynaecologists (RCOG) consider there is currently not enough evidence to recommend this surgery and advocate further research to fully evaluate the procedure [18–20].

Centred on the above reason, we decided to conduct a meta-analysis to evaluate the reproductive outcomes after hysteroscopic septum resection in women with septate uterus compared to women who opted for conservative management. Our aim was to determine whether hysteroscopic septum resection improves reproductive outcomes in women with septate uterus and previous adverse reproductive outcomes.

## Materials and methods

This study was performed and reported according to the criteria of Preferred Reporting Items for Systematic Reviews and Meta-analyses (PRISMA) [21]. We performed a comprehensive systematic review and meta-analysis to evaluate the efficacy of hysteroscopic septum resection on the reproductive outcomes of patients with uterine septum and a history of subfertility and/or previous poor reproductive outcomes. A search was conducted on the International Prospective Register for Systematic Reviews (PROSPERO) to ensure there were no systematic reviews and meta-analyses similar to this which had been recently published or were in the process of being conducted; none were found. The PROSPERO registration number is  CRD42021227035.

### Literature search

**Fig. 1:**
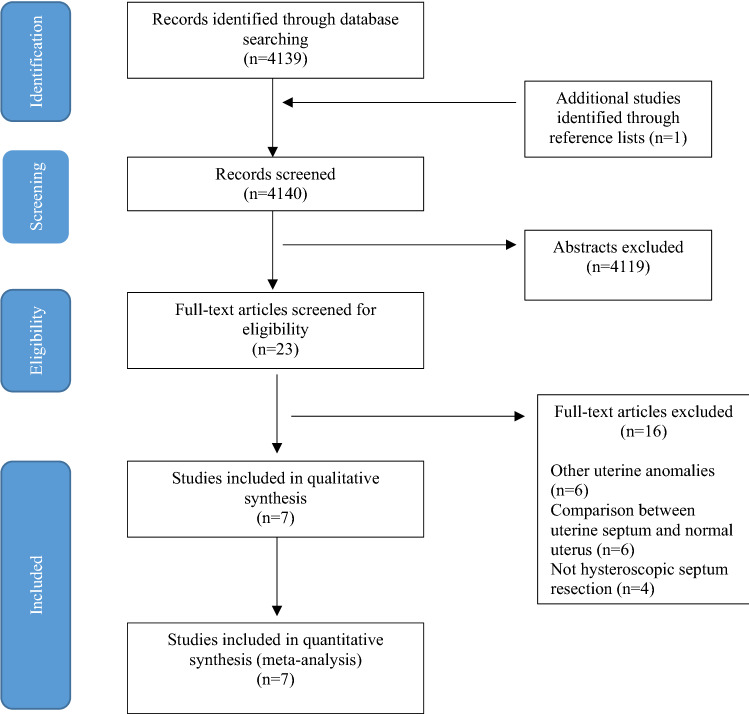
Flow chart of literature search and data extraction

AMED, BNI, CINAHL, EMBASE, EMCARE, Medline, PsychInfo, PubMed, Cochrane register of controlled trials (CCTR), Cochrane database of systematic reviews (CDSR) and CINAHL were searched from inception up to April 2020 for studies that looked at reproductive outcomes following hysteroscopic septum resection. A combination of search and MeSH terms were employed including “uterine septum”, “uterine anomaly/uterine anomalies”, “metroplasty”, “reproductive outcome”, “septal resection”, “uterine malformation”, “septate uterus” and “hysteroscopy”. A manual search of reference lists of all known and included studies was conducted to identify studies not captured by electronic searches (Fig. 1).

The title and abstracts were screened by two independent reviewers (MK and BN). Full articles of all citations which were likely to meet the predefined selection criteria were obtained.

### Eligibility criteria

Relevant randomised controlled trials and comparative studies were considered eligible for this review; case reports were excluded. We only included studies which compared outcomes in women with uterine septum suffering from subfertility or poor reproductive outcomes who were treated by hysteroscopic septum resection with women not treated, and provided data on clinical pregnancy rates, miscarriage rate and obstetric outcomes.

As this study aimed to examine the effect of uterine septum, studies that included other uterine anomalies were excluded to avoid potential bias. Women with complete septate uterus with both duplicated cervix and vaginal septum were also included as vaginal septum is not known to adversely affect reproductive outcomes. Studies with outcome data in the same women before and after treatment were excluded.

### Participants

Participants included women with uterine septum who suffered from subfertility or previous poor reproductive outcomes and were attempting to conceive with or without hysteroscopic septum resection.

### Outcome measures

The primary outcome of the study was live birth rate. A priori determined secondary outcomes were included in this meta-analysis including clinical pregnancy rate (pregnancy rate per patient) in women treated versus with those women not treated. Likewise, probability of miscarriages, preterm labour and malpresentations were compared with women treated with those not treated were assessed as secondary endpoints.

### Data extraction

Data was extracted independently by two authors (MK and BN) and recorded on a data collection form. Any discrepancies were settled by discussion with the senior author (MM). For each eligible study, data regarding demographics (citation data, country, study period, number of patients included), methodology (retrospective or prospective), population of the study (regarding both ‘cases’ and ‘control’ groups), uterine anomalies evaluated, mode of diagnosis, follow-up period, clinical pregnancy rate, miscarriage rate, live birth rate, preterm delivery, term delivery and malpresentations was collected.

### Quality, risk of bias and publication bias assessment of included studies

**Fig. 2:**
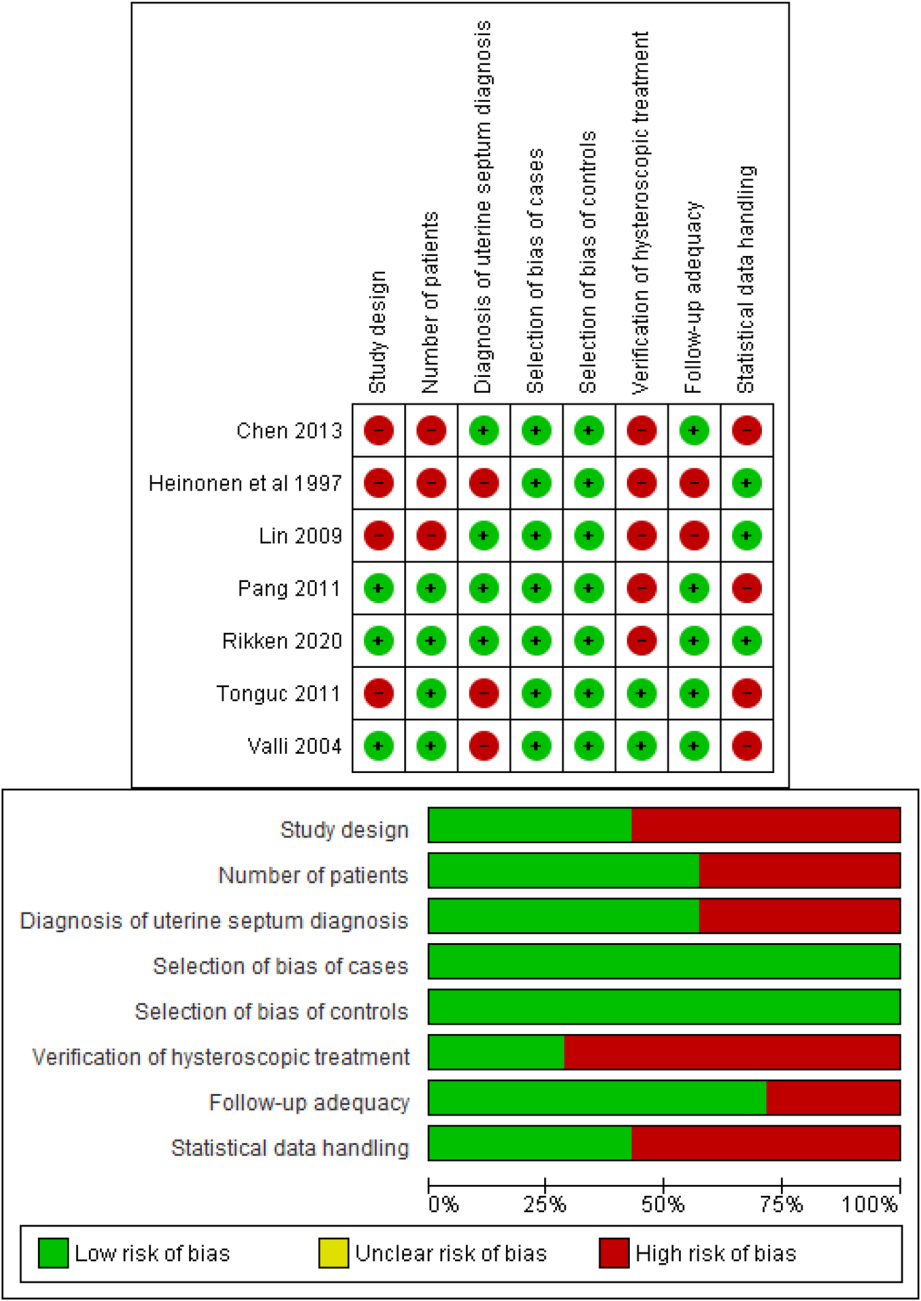
**a.** Risk of bias graph for each of the studies included in this systematic review (modified Newcastle–Ottawa scale for observational studies), **b.** overall bias risk assessment which suggests relatively high risk at the time of verifying hysteroscopic treatment and handling the data statistically

**Fig. 3:**
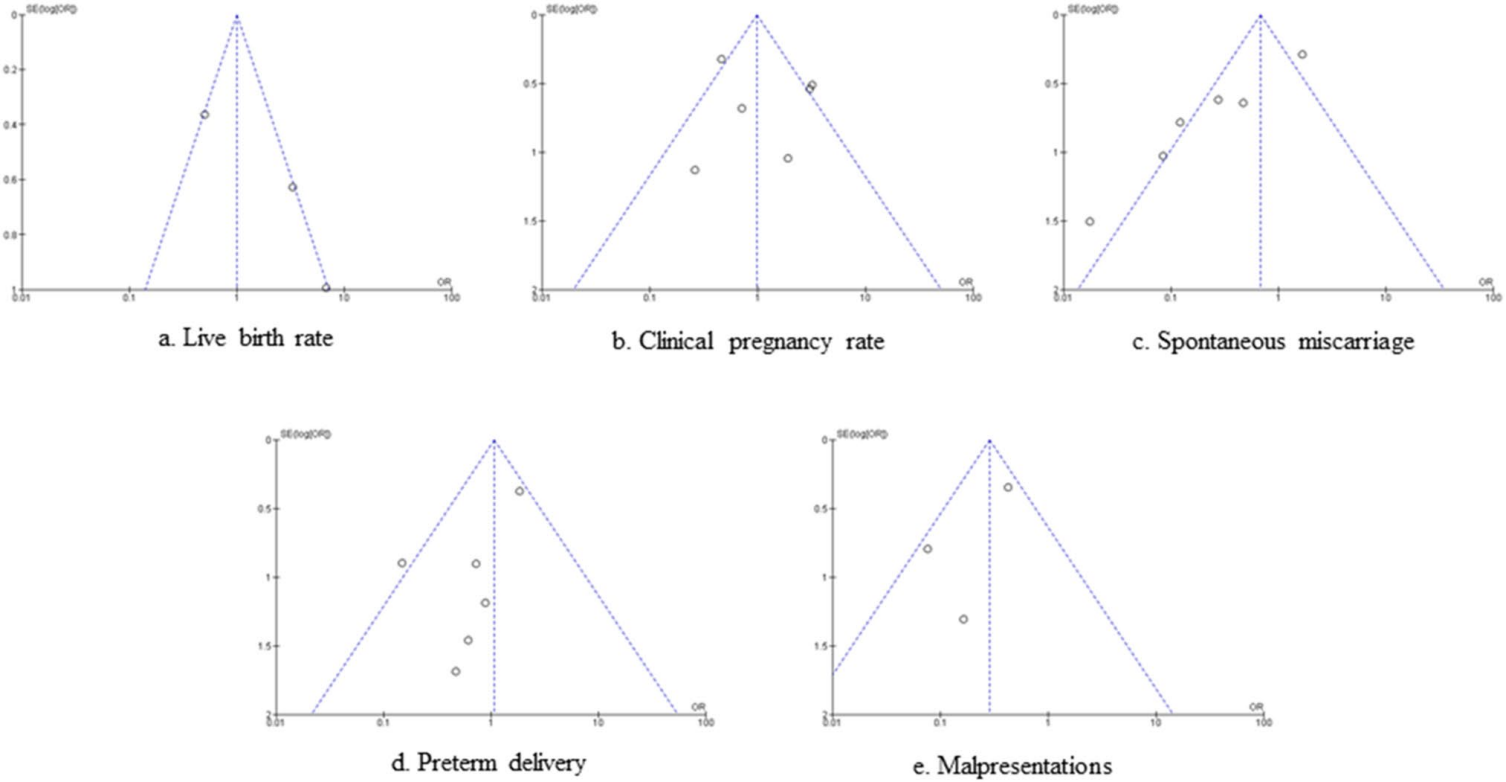
Funnel plot of the meta-analysis of the published studies for **a.** Live birth rate, **b.** clinical pregnancy rate, **c.** Spontaneous miscarriage, **d.** Preterm delivery and **e.** Malpresentations. The triangle lines represent were region, where 95% of the data points (effect size/ sample size) would lie in the absence of publication bias

For bias risk assessment, the Newcastle–Ottawa scale for assessment of non-randomised studies was used, based on the recommendation of the Cochrane Collaboration [22, 23] and adapted for the specific research questions [24]. The following items were considered for quality assessment: (i) whether the study design was prospective; (ii) number of patients; (iii) whether the diagnosis of uterine septum was accurate; (iv) selection bias of cases and controls; (v) verification of hysteroscopic treatment (e.g. second look hysteroscopy); (vi) adequacy of follow-up and (vii) whether studies used matching and/or multivariate method to control for potential effect of confounders (Fig. 2; Supplementary Tables 1 and 2). Publication bias was assessed using funnel plots (Fig. 3).

### Statistical analysis

Dichotomous data were extracted from the individual studies and expressed as combined odds ratio (OR) with 95% confidence intervals (CI) using the Review Manager 5.3 software (RevMan version 5.3; The Nordic Cochrane Centre, Copenhagen, The Cochrane Collaboration, 2014).

Results of the Chi-squared and *I*^*2*^ statistics were used to determine statistical heterogeneity. An *I*^*2*^ statistic with a value greater than 50% or a chi-squared statistic that was larger than its degree of freedom was interpreted as significant heterogeneity between studies. Where there was evidence for significant statistical heterogeneity, a random effects model was used for meta-analysis; otherwise, a fixed effects model was used. A sensitivity analysis was performed by excluding studies of low-quality on the adapted Newcastle–Ottawa scale.

## Results

### Systematic review and characteristics of included studies

The literature search yielded 4139 publications in total and an additional one study was identified through reference lists. The titles and abstracts of these manuscripts were screened, resulting in 23 studies considered potentially eligible for the review. Of the total 23 potentially relevant manuscripts identified, 16 studies were excluded after evaluating the full text. The reasons of exclusion include other uterine anomalies (*n* = 6), outcome comparison between septate uterus and normal uterus (*n* = 6), other techniques for septum resection apart from hysteroscopic resection (*n* = 4). A total of seven studies [25–31] involving 659 women, 407 who had hysteroscopic septum resection and 252 who did not, were included in the meta-analysis (Fig. 1; Table 1). No randomised controlled trials were identified, only observational studies. All selected studies were published between 1997 and 2020, and only two studies were fully conducted in a prospective design, whereas one study was part prospective and part retrospective.

**Table 1:** Characteristics of included studies

Authors	Country	Data type	Study period	Sample size (*n* =)	Surgery vs no surgery (*n* =)	Type of surgery	Study population	Anomaly	Septate uterus criteria/ diagnostic method	Outcome measures	Follow-up (months)
Chen et al. [25]	Guangzhou, China	Retrospective	1997–2010	21	Surgery Yes = 11 No = 10	Not described	History of infertility or previous poor reproductive outcomes	Complete septate uterus with both duplicated cervix and vaginal septum	Vagina, cervix, uterus, adnexa-associated malformation (VCUAM) classification system/Transvaginal USS, hysterotomy during C-section and hysteroscopy	Pregnancy rate, preterm labour, abnormal presentation, ongoing pregnancy, spontaneous miscarriage, mode of delivery	6–24
Heinonen et al*.* [28]	Tampere, Finland	Retrospective	1962–1995	38	Surgery Yes = 19 No = 19	Semi-rigid scissors and rectoscope	History infertility or previous poor reproductive outcomes	Septate and subseptate uterus	Proposed classification [32]/Diagnostic method not described	Pregnancy rate, abortion, preterm delivery, term delivery	Not described
Lin et al*. *[26]	Zhejiang, China	Retrospective	1998–2007	35	Surgery Yes = 20 No = 15	Metzenbaum scissors	History infertility or previous poor reproductive outcomes	Complete septate uterus with both duplicated cervix and vaginal septum	Classification not described/Cross-sectional 3D USS and hysterosalpingography	Number of pregnancies, rates of spontaneous and induced abortion, rates of preterm and term deliveries, rates of operative deliveries, live birth, adherent placenta and uterine ruptures	18
Pang et al. [27]	Nanning, China	Prospective	2006–2011	138	Surgery Yes = 46 No = 32	Not described	At least two previous miscarriages or no previous poor reproductive outcomes	Subseptate uterus	American Society for Reproductive Medicine guidelines/3D USS	Rate of pregnancy, spontaneous abortion, preterm delivery, full term delivery	15
Rikken et al. [29]	18 centres Netherlands, 2 centre USA, 1 centre UK	Retrospective (+ Prospective- In Netherlands)	2000–2018	257 women (123 retrospective and 20 prospective)	Surgery Yes = 151 No = 106	73 versa point, 32 with scissors, 12 with electrosurgery, 34 unknown	History of infertility or previous poor reproductive outcomes	Uterine septum	Classification system at that time/HSG, 3D-US, MRI saline or gel infusion sonohysterography or hysteroscopy combined with laparoscopy	Primary—live birth (> 24 weeks), Secondary—ongoing pregnancy, early pregnancy loss, preterm birth and foetal malpresentation	40 for septum resection, 53 for no surgery
Tonguc et al. [30]	Ankara, Turkey	Retrospective	2006–2009	127	Surgery Yes = 102 No = 25	Monopolar 90° angle knife electrode	Primary infertility	Uterine septum	American Society for Reproductive Medicine guidelines/Vaginal and abdominal USS and office hysteroscopy and hysterosalpingography	Pregnancies, abortions, preterm delivery, term delivery, live birth rate	14 after surgery, 14 after normal hysteroscopy in surgery
Valli et al. [31]	Tor Vergata, Italy	Prospective	1990–2001	43	Surgery Yes = 28 No = 15	Resectoscope loop at 80–100 W for cutting	At least two previous miscarriages	Septate uterus	American Society for Reproductive Medicine guidelines/Diagnostic hysteroscopy	Pregnancies, term pregnancies, preterm, abortion	36

*HSG* hysterosalpingogram, *MRI* magnetic resonance imaging, *USS* ultrasound

The population in each study varied regarding the research question addressed in each study (Table 1). Some studies evaluated the potential benefits of hysteroscopic septum resection on infertile women (*n* = 1), in patients with a history of recurrent miscarriages (*n* = 2) and in women with either subfertility or previous poor reproductive outcomes (*n* = 4). The study by Pang et al. [27], for example, analysed two subpopulations of patients: women who had experienced recurrent spontaneous abortion and women with no history of poor reproductive outcome. Only the women with the recurrent spontaneous abortion were included in the meta-analysis. The study by Heinonen et al*.* [28] looked at other uterine anomalies and abdominal metroplasty together with hysteroscopic septum resection. However, in the meta-analysis only the data from women who underwent hysteroscopic septum resection compared with match-controlled women with uterine septum without surgery were included. Certain studies included women with a specific type of uterine septum, whereas others included a broader classification (Table 1). The classification system used for the definition of uterine septum was also variable in each study, although the majority of the studies favoured the American Society of Reproductive Medicine guidelines [3]. The diagnosis of uterine septum also varied between studies. Three-dimensional ultrasound alone was used in one study, hysteroscopy alone in another and previous surgical records alone in a third study. One study did not mention the method of diagnosis of uterine septum. Multiple methods were used to screen and confirm the presence of uterine septum in the remaining studies (Table 1). Most studies were of acceptable quality, although one study [28] was considered low quality based on the Newcastle–Ottawa scale (Fig. 2; Supplementary Tables 1 and 2). The sub-arm within the study by Pang et al*.* [27], in which participants had no previous reproductive issues, was not included in the meta-analysis.

### Synthesis of results

**Fig. 4:**
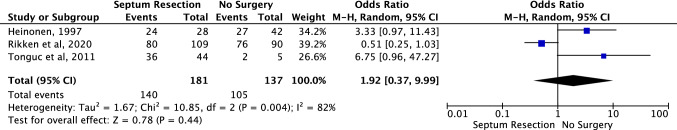
Effect of hysteroscopic uterine septum resection on live birth

Live birth rate (Fig. 4): Only three studies looked at live birth rate; a pooled analysis was performed on those three studies with a total of 181 patients who had hysteroscopic septum resection and 137 patients who were managed without septum resection [28–30]. The analysis found no evidence of a significant difference in live birth rate between the groups (OR 1.92, 95% CI 0.37–9.99). There was, however, evidence of significant heterogeneity amongst the included studies (chi-squared = 10.85, df = 2, *P* = 0.44, *I*^*2*^ = 82%).

**Fig. 5:**
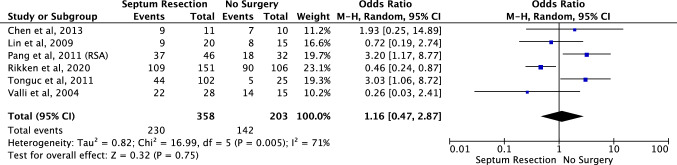
Effect of hysteroscopic uterine septum resection on clinical pregnancy rate

Clinical pregnancy rate (Fig. 5): For the analysis of clinical pregnancy rate, a pooled analysis was performed on six studies with a total of 358 patients who had hysteroscopic septum resection and 203 patients who were managed without septum resection [25–27, 29–31]. One study was excluded from the meta-analysis [28] as the index pregnancy could not be determined from the results provided. The analysis found no evidence of a significant difference in clinical pregnancy rate between the groups (OR 1.16, 95% CI 0.47–2.87). Evidence of significant heterogeneity was found amongst the included studies (chi-squared = 16.99, df = 5, *P* = 0.75, *I*^*2*^ = 71%).

**Fig. 6:**
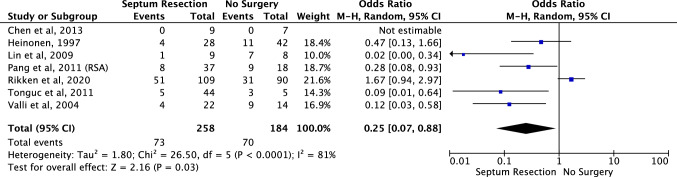
Effect of hysteroscopic uterine septum resection on spontaneous miscarriage

Spontaneous miscarriage (Fig. 6): All seven studies looked at spontaneous miscarriage with 258 women having had the hysteroscopic septum resection compared with 184 women who did not [25–31]. The analysis found a significantly lower miscarriage rate in women who had hysteroscopic septum resection (OR 0.25, 95% CI 0.07–0.88) compared to those who had opted for conservative management. Similarly, to previous outcomes, there was evidence of significant heterogeneity amongst the included studies (chi-squared = 26.50, df = 5, *P* = 0.03, *I*^*2*^ = 81%).

**Fig. 7:**
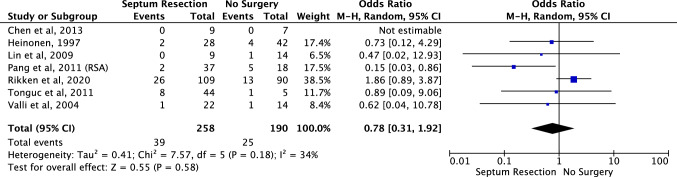
Effect of hysteroscopic uterine septum resection on preterm delivery

Preterm delivery (Fig. 7): All seven studies looked at preterm delivery with 258 women having had the hysteroscopic septum resection compared with 190 women who did not [25–31]. No significant difference was found in preterm delivery between the groups (OR 0.78, 95% CI 0.31–1.92). There was evidence of significant heterogeneity amongst the included studies (chi-squared = 7.57, df = 5, *P* = 0.58, *I*^*2*^ = 34%).

**Fig. 8:**
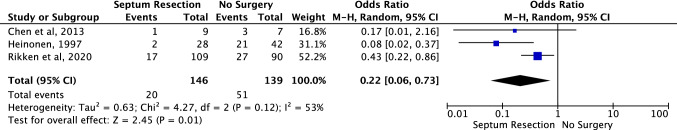
Effect of hysteroscopic uterine septum resection on malpresentations

Malpresentations (Fig. 8): Only three studies looked at malpresentation rate. A pooled analysis was performed on these three studies which included a total of 146 patients who had hysteroscopic septum resection and 139 patients who were managed without septum resection [25, 28, 29]. The analysis found a significantly lower rate of malpresentations in women who had hysteroscopic septum resection (OR 0.22, 95% CI 0.06–0.73). Significant heterogeneity was also identified amongst the included studies (chi-squared = 4.27, df = 2, *P* = 0.01, *I*^*2*^ = 53%).

### Sensitivity analysis

One study [28] was deemed low quality from the adapted Newcastle–Ottawa scale and was, therefore, excluded from the sensitivity analysis. For the analysis of live birth rate, a pooled analysis was performed on two studies [29, 30] which included 153 women following hysteroscopic septum resection and 95 women treated conservatively. The sub-analysis still showed no significant difference between the two groups (OR 1.57, 95% CI 0.13–19.40). However, heterogeneity across the studies still remained high (chi-squared = 5.99, df = 1, *P* = 0.73, *I*^*2*^ = 83%).

For the analysis of miscarriage rate, a pooled analysis was performed on six studies [25–27, 29–31] which comprised 230 patients following hysteroscopic septum resection and 142 patients without septum resection. Results continued to show a significant decrease in miscarriage rate following hysteroscopic septum resection (OR 0.20, 95% CI 0.04–0.99). Nevertheless, no improvements were seen in heterogeneity between the studies (chi-squared = 26.04, df = 4, *P* = 0.05, *I*^*2*^ = 85%).

For the analysis of preterm delivery, a pooled analysis was performed on six studies [25–27, 29–31] including 230 patients following hysteroscopic septum resection and 148 patient without septum resection. The analysis once again showed no difference in preterm delivery between the groups (OR 0.72, 95% CI 0.23–2.27) or on the high degree of heterogeneity between the studies (chi-squared = 7.34, df = 4, *P* = 0.57, *I*^*2*^ = 46%).

For the analysis of malpresentations, a pooled analysis was performed on two studies [25, 29] which included 118 women following hysteroscopic septum resection and 97 women managed conservatively. Similarly, to the original findings, the subgroup analysis showed a significant decrease in the risk of malpresentations following hysteroscopic septum resection (OR 0.40, 95% CI 0.21–0.79). Furthermore, a significant decrease in heterogeneity between the studies was noted (chi-squared = 0.49, df = 1, *P* = 0.007, *I*^*2*^ = 0%).

### Publication bias

The funnel plots for the primary outcome and almost all secondary endpoints were rather symmetric showing little evidence of publication bias (Fig. 3).

## Discussion

This systematic review and meta-analysis provide an up-to-date review of the available literature and summarises the evidence on the highly controversial topic of hysteroscopically resecting the uterine septum in women with previous history of infertility and/or adverse reproductive outcomes.

The first and foremost finding of this study was a significant decrease in the risk of miscarriage after uterine septum resection. The study, however, did not find any significant evidence to suggest hysteroscopic resection improves live birth and clinical pregnancy rates, and/or reduces preterm delivery. As expected, our review also confirmed that uterine septum resection is associated with a significant decrease in malpresentation during labour.

To the best of our knowledge this is the first review and meta-analysis which, thoroughly and systematically, compares reproductive outcomes of hysteroscopic uterine septum resection versus conservative management in patients with history of subfertility and/or previous poor reproductive outcomes. Partial attempts to critically appraise the available literature on this issue have been made in the past. Venetis et al. [24] conducted a meta-analysis looking at congenital uterine anomalies and concluded that the hysteroscopic septum resection may have beneficial effects on reproductive outcomes in women with uterine septum in terms of decreasing the rate of spontaneous miscarriage. Furthermore, they also suggested that hysteroscopic septum resection may improve achievement of a successful pregnancy and reduction of preterm delivery [24]. We believe that some of the discrepancies between their findings and our results could be multifactorial. Firstly, Venetis et al*.* [24] included women from the study by Pang et al*.* [27] sub-arm with no previous poor reproductive outcomes. On the contrary, we purposely excluded these patients to focus only those with subfertility or previous adverse reproductive history. Furthermore, at least two additional studies have been published, since the publication of Venetis et al*.* work, which we have included in this meta-analysis [25, 29]. Finally, whereas Venetis’s meta-analysis only looked at clinical pregnancy rate, preterm birth and miscarriage, we also evaluated equally relevant reproductive outcomes such as live birth rate and malpresentations.

Nonetheless, our study does not come without a series of limitations. The main drawback we found was the quality of the primary studies which were in majority noted to be small sized studies, mostly retrospective and not adjusted for confounding factors. Only a limited number of studies took individual measures (e.g., multivariate analyses or matching procedures) against known confounders (e.g., body mass index and age) bias. In the study by Rikken et al. [29] in which septum resection did not lead to improved reproductive outcomes compared with expectant management, for example, 19 women who chose against surgery were pregnant at the time of diagnosis of uterine septum compared with none of the women who underwent hysteroscopic septum resection. Such a difference between control-cases is likely to have skewed and biased the outcomes.

Our review also highlights significant heterogeneity in the available literature. Eligible studies were conducted across a span of 20 years (1997–2020) during which system classifications and surgical techniques dramatically changed. Furthermore, studies varied on the criteria they employed to classify uterine septum, outcome measures and define follow-up periods. We are aware that this relatively high heterogeneity amongst the studies is likely to affect some of our findings. In an attempt to compensate the high heterogeneity in the primary data, we used a random-effect model, which acknowledges that the sample analysed in the different studies might not all originate from the same population.

Based on the modified Newcastle–Ottawa quality assessment scale, which was specifically adapted for this meta-analysis, six out of the seven studies were graded high or average quality with only one study being low quality. To account for this, we performed a sensitivity analysis excluding the low-quality study. Removing the low-quality study from the meta-analysis did not significantly alter our results, and only reduced heterogeneity for the malpresentation secondary outcome in which *I*^*2*^ dropped from 53 to 0%.

It is worth noticing though that most of the included studies did not account for the size of the uterine septum. Septum size could have influenced the reproductive outcomes and possibly led to confounding by indication. Furthermore, the uterine septum size could have influenced the choice of treatment by the patient and/or their physician. We also noted that in most of the studies in this review, the group was self-selected between treatment and expectant management [26, 27, 30, 31].

Overall, this systematic review shows potential benefit of hysteroscopically resecting the uterine septum in women with previous history of infertility and/or adverse reproductive outcomes to reduce miscarriage and malpresentation in labour. However, our study also highlights the need for larger, fully powered, prospective trials before further clinical conclusions regarding hysteroscopic septum resection can be made. We hope the awaited results from Netherlands Trial Register (NTR) 1676 will cast some further light on the matter.

## Conclusion

Our systematic review and meta-analysis demonstrate that hysteroscopic removal of uterine septum in women with subfertility or previous poor reproductive outcomes reduces the probability of miscarriage and malpresentations. However, it does not seem to significantly affect live birth rate, pregnancy achievement and preterm delivery even though these findings should be interpreted with caution given the limited evidence available.

In that respect, our study highlights substantial gaps in high-quality evidence regarding the value of hysteroscopic uterine septum resection on relevant clinical reproductive outcomes, and it supports the need for larger, fully powered, multicentre trials which can help better counsel and guide clinical management of women with uterine septum unable to achieve a successful pregnancy at term.

## Supplementary Information

Below is the link to the electronic supplementary material.

Supplementary file1 (DOCX 15 KB)
