# Supplementary material for: Does hysteroscopic resection of uterine septum improve reproductive outcomes: a systematic review and meta-analysis
Source: Arch Gynecol Obstet. 2021 Feb 7;303(5):1131–42. doi: 10.1007/s00404-021-05975-2 (PMC8053150; doi:10.1007/s00404-021-05975-2)
Supplement: Supplementary file 1 — Supplementary file1 (DOCX 15 KB) [file 404_2021_5975_MOESM1_ESM.docx]

**Supplementary Table 1.**  Newcastle–Ottawa scale for quality and risk of bias assessment of included studies adapted for the specific research questions [23]

| **Study** | **Study Design** | **Number of patients** | **Diagnosis of uterine septum accuracy** | **Selection bias of cases** | **Selection bias of controls** | **Verification of hysteroscopic treatment** | **Follow-up Adequacy** | **Statistical Data Handling** | **Total** |
| --- | --- | --- | --- | --- | --- | --- | --- | --- | --- |
| **Chen *et al.,* 2013** | - | - | * | * | * | - | * | - | 4/8 |
| **Heinonen *et al.,***  **1997** | - | - | - | * | * | - | - | * | 3/8 |
| **Lin *et al*., 2009** | – | – | * | * | * | – | – | * | 4/8 |
| **Pang *et al*., 2011** | * | * | * | * | * | – | * | – | 6/8 |
| **Rikken *et al.,* 2020** | * | * | * | * | * | - | * | * | 7/8 |
| **Tonguc *et al*.,**  **2011** | – | * | – | * | * | * | * | – | 5/8 |
| **Valli *et al*.,**  **2004** | * | * | – | * | * | * | * | – | 6/8 |

*Classification of studies: high-quality studies: >5; average quality studies: 4–5; Low quality studies: 0–3.*

**Supplementary Table 2.**  Coding scheme of the study quality assessment

| **Study design** | 1 star for prospective design |
| --- | --- |
| **Number of patients** | 1 star for studies with ≥ 25 cases |
| **Diagnosis of uterine septum accuracy** | 1 star for studies that use a combination of hysteroscopy/laparoscopy, sonohysterography or three-dimensional ultrasound |
| **Selection bias of cases** | 1 star when selection bias not evident |
| **Selection bias of controls** | 1 star when selection bias not evident |
| **Verification of hysteroscopic treatment of septum** | 1 star for second look hysteroscopy to confirm adequacy of hysteroscopic septum resection |
| **Follow-up adequacy** | 1 star for follow-up of ≥ 12 months |
| **Statistical data handling** | 1 star when study used matching or multivariate method to control for potential effect of confounders |
